# Strengthened PAN-based carbon fibers obtained by slow heating rate carbonization

**DOI:** 10.1038/srep22988

**Published:** 2016-03-23

**Authors:** Min-A Kim, Dawon Jang, Syogo Tejima, Rodolfo Cruz-Silva, Han-Ik Joh, Hwan Chul Kim, Sungho Lee, Morinobu Endo

**Affiliations:** 1 Carbon Convergence Materials Research Center, Korea Institute of Science and Technology, San 101 Eunha-ri, Bongdong-eup, Wanju-gun, Jeonbuk 565-905, Korea; 2 Department of Organic Materials and Fiber Engineering, Chonbuk National University, 567 baeje-daero, Deokjin-gu, Jeonju, Jeonbuk 561-756, Korea; 3 Department of Nanomaterials Engineering, Korea University of Science and Technology,217 Gajeong-ro, Tuseong-gu, Daejeon, 305-350 Korea; 4 Global Aqua Innovation Center, Shinshu University, 4-17-1 Wakasato, Nagano 380-8553, Japan; 5 Institute of carbon science and technology, Shinshu University, 4-17-1 Wakasato, Nagano 380-8553, Japan; 6 Research Organization for Information Science & Technology, 2-32-3, Kitashinagawa, Shinagawa-ku, Tokyo, 140-0001, Japan

## Abstract

Large efforts have been made over the last 40 years to increase the mechanical strength of polyacrylonitrile (PAN)-based carbon fibers (CFs) using a variety of chemical or physical protocols. In this paper, we report a new method to increase CFs mechanical strength using a slow heating rate during the carbonization process. This new approach increases both the carbon sp^3^ bonding and the number of nitrogen atoms with quaternary bonding in the hexagonal carbon network. Theoretical calculations support a crosslinking model promoted by the interstitial carbon atoms located in the graphitic interlayer spaces. The improvement in mechanical performance by a controlled crosslinking between the carbon hexagonal layers of the PAN based CFs is a new concept that can contribute further in the tailoring of CFs performance based on the understanding of their microstructure down to the atomic scale.

Extensive research of PAN-based carbon fibers (CFs) has focused on developing new precursors and modifying the process of stabilization and carbonization in order to control and to maximize their mechanical performance^1^,^2^,^3^,^4^. Particularly, a number of steps and temperature variations implemented to reach the final carbonization and graphitization temperatures have been recognized as important variables^5^. It is well-known that increasing the final carbonization temperature up to ~1200 °C increases both the tensile strength and the modulus of CFs, but a further temperature increase can affect the tensile strength while still increasing the tensile modulus because the carbon microstructure changes to a more graphitic one^6^. Therefore, during the production of high-strength grade CFs, the maximum temperature for carbonization is generally below 1200 °C. Another important feature of the CFs production process is the two-step carbonization process of the PAN precursor; the first step is a low-temperature treatment (LTT) at 600–800 °C, while the second step is a high-temperature treatment (HTT) at ~1200 °C (5–10 min for each step)^7^. It should be noticed that the evolution of gas during the carbonization process must be taken into account in order to optimize the tensile strength. Indeed, the carbonization stage is a process where elements such as nitrogen, oxygen, and hydrogen are removed from the precursor fibers, leaving a material with at least 93 wt% of carbon content^7^,^8^.

We herein report the effect of the microstructure in PAN-based CFs on their mechanical properties. Commercially available PAN fibers were stabilized by oxidation in air using a tailor-made apparatus. The resulting fibers were infusible and could be carbonized in a tubular furnace at temperatures up to 1000, 1050, 1100, and 1200 °C with heating rates of 0.5–10 °C/min, respectively. The chemical compositions of the CFs, particularly the carbon and nitrogen content, and the chemical environment as a function of the carbonization temperature and heating rate were correlated with mechanical properties. We also carried out MD simulations to understand the role of nitrogen species and modeled the carbon sp^3^ structure. We found that both the sp^3^ hybridized carbon and especially the quaternary nitrogen atoms have a strong effect on the tensile strength of the fibers. We have proposed a microstructure model where two adjacently stacked hexagonal carbon networks strongly bond by interstitial sp^3^ carbons with the aid of the interlayer forces, which are enhanced by the neighboring quaternary nitrogen at the bonding site. This model of carbon microstructure is a new concept in the high-strength CF research field. This research is expected to contribute significantly to the production of carbon fiber for high performance products.

## Materials and Methods

### Preparation of sample

Commercial as-spun PAN-based (>95% polyacrylonitrile) fibers obtained from Jilin Company Co., LTD were stabilized using the tailor-made continuous stabilization apparatus at temperatures ranging from 195 to 280 °C for 120 min under ordinary atmosphere. After stabilization, the samples were carbonized at 1000, 1050, 1100, and 1200 °C, respectively, using a tube furnace (Korea Furnace Development Co., Ltd) under nitrogen gas (purity 99.999%, Korean Gas Co. ltd) atmosphere (flow rate of 2000 cc/min) at a heating rate ranging from 0.5 to 10 °C/min (from room temperature up to each carbonization temperature).

### Characterizations on the resultant PAN-based carbon fibers

The mechanical properties of the CFs were examined by a mechanical tester for a single fiber (FAVIMAT+, Textechno, Germany) with a test speed of 2 mm/min. The gauge length was 25 mm, and 20 specimens were measured for each experimental point.

TEM (Tecnai G2 F20, FEI) was used to investigate the crystalline structure of CFs using an acceleration voltage of 200 kV. To confirm the crystalline structure of the carbon fiber, the CFs were dispersed in ethanol using deep sonication and then placed on a carbon film-supported copper grid and dried in a vacuum oven at 60 °C. FFT analysis was conducted on the TEM images.

X-ray photoelectron spectroscopy (XPS, K-alpha, Thermo Scientific, USA) was performed using monochromated Al Kα (1486.6 eV) X-rays to examine the type of chemical bond of nitrogen and the elemental composition on the surface of CFs samples. The survey spectrum was collected from 0 eV to 1350 eV, and the binding energies were referenced to the C 1s line at 284.8 eV.

Raman spectroscopy was carried out by a Raman spectrometer (LabRAM HR, Horiba Jobin Yvon, France) to determine the layer length empirically and observe the development of the carbon structure of the fibers^9^,^10^. Raman spectra were measured in the range of 800–3500 cm^−1^ in continuous scanning mode under a laser excitation wavelength of 514 nm and a power of 16 mW. The laser beam was focused by a 50× objective lens resulting in a spot size of approximately 1 μm in diameter. The acquisition time and the number of circulations were 10 s and 3 times, respectively.

X-ray diffraction analysis (XRD, SmartLab, Rigaku, Japan) was performed to investigate the crystalline structure of CFs and calculate the interlayer spacing d_002_ (Å) by Cu Kα radiation (λ=1.54 Å). The acceleration voltage and emission current were 45 kV and 200 mA, respectively. The 2θ value ranged from 10 to 60°, and the scan speed was 4°/min.

The degree of crystalline orientation, the preferred orientation of the crystalline c-axis, was determined using wide angle X-ray diffraction analysis (WAXD, D8 DISCOVER, Bruker, USA). The samples were exposed to the X-ray beam for 100 s and the operating voltage and current were 40 Kv and 40 mA, respectively.

Elemental analysis (Flash 2000, Thermo Scientific, USA) was carried out to measure the amounts of C, H, N, S and O in the bulk of the CFs samples. The elements of C, H, N and S were analyzed using oxygen and helium gas at 900 °C for 720 s. Oxygen was analyzed using helium gas at 1060 °C for 500 s.

### Determine of crystalline parameters

La was determined using following equation (1) developed by Knight and White^10^:


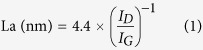


where I_D_ and I_G_ are intensities of D and G bands from Raman spectrum.

The values of d_002_ and the Lc were calculated using the Bragg’s law and Deby-Scherrer formula, respectively^11^,^12^:


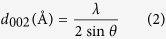



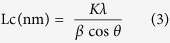


where λ, θ, K, and β are the wavelength of the x-ray source from Cu (1.54 Å), the diffracted angle, shape factor (normally 0.9), and the full width at half maximum in radians, respectively.

The degree of crystalline c-axis orientation was calculated using the following equation^13^:


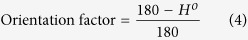


where H is the full width at half maximum in radians.

### MD simulations

From the theoretical point of view, the *ab initio* atomic calculations were performed using density functional theory (DFT). The code we used is the Plane-Wave Self-Consistent Field (PWscf) program in the Quantum Espresso packages^14^. The Vanderbilt ultrasoft pseudopotential was applied for the ionic effective potential, and the generalized gradient approximation functional parameterization of Predew Burke and Ernzerhof was applied for the exchange-correlation energy^15^,^16^. The dispersion interaction of van der Waals (vdW) type with a non-local functional is considered as vdW-DF for the exchange-correlation in the PWscf calculation^17^. Our calculations were done within only a gamma point for the k-point sampling and a plane-wave cutoff energy of 544 eV. The atomic structural optimization, called structure relaxation, was calculated in order to study the origins of the binding interaction between twisted carbon hexagonal networks or so-called turbostratic stacking structure “with and without” quaternary nitrogen atoms in the networks and an interstitial C atom between layers. Both upper and lower layers consist of 48 atoms, respectively, and the size of the layer is 7.36 Å × 11.39 Å.

## Results

**Figure 1:**
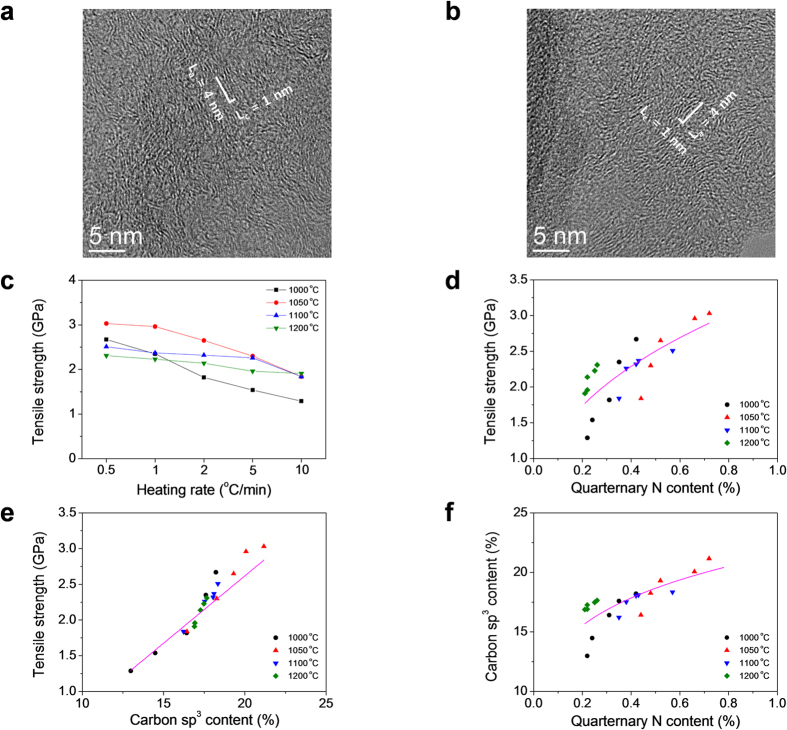
(**a**,**b**) TEM images of CFs heated 1050 °C with heating rates of (**a**) 0.5, and (**b**) 10 °C/min. (**c**) Tensile strength of CF as a function of the heating rate at carbonization temperatures are shown. Relationships between tensile strength and (**d**) quaternary N atomic content, and (**e**) carbon sp^3^ content in total CF. (**f**) Relationships between quaternary N atomic content and carbon sp^3^ content in total CF.

Crystalline dimension values ranging from 4–5 nm, 1–1.3 nm, and 0.35–0.36 nm for L_a_, L_c_, and d_002_, respectively (Figure S4a–c), were determined from Raman spectra and X-ray diffraction, as shown in Figures S1 and S2. The relative crystalline orientation of CFs determined from XRD was found to be 0.72–0.74°, i.e., their c-axis is almost perpendicular to the fiber axis (Figure S3). Figure 1a,b show the HR-TEM micrographs of two different CFs carbonized at 1050 °C using two different heating rates: 0.5 and 10 °C/min. Interestingly, both fibers showed similar crystallite dimensions and microstructure regardless of the different heating rates. In general, the structural information shows that different ultimate carbonization temperatures and heating rates did not affect the microstructure of the resulting CFs. All samples showed similar orientation and size of the crystallites. Similarly, the fiber diameters and surface morphology were similar regardless of the processing conditions. All fibers showed the typical kidney-shaped cross section characteristic of PAN-based CFs (Figure S5). Nevertheless, the mechanical properties, as shown later, did change considerably as a function of the heat treatment parameters, suggesting that a mechanism that is more complex than one considering just the crystallite size and orientation lies behind the mechanical performance of CFs.

The changes in the heat treatment protocol significantly modified the tensile properties of the different CFs, as shown in Fig. 1c. When the ultimate carbonization temperature was 1050 °C, the tensile properties increased monotonously when decreasing the heating rate from 10 to 0.5 °C/min, changing the tensile strength from 1.8 to 3.0 GPa. A further increase in the ultimate heating temperature to 1200 °C led to a decrease of the tensile strength. The strain to failure did not show any significant differences, and it had values between 1.1 and 1.5% depending on the heat treatment condition except for the sample carbonized at the lowest temperature (1000 °C) (Figure S6). These results indicate that a higher temperature of carbonization not only leads to a general decrease of the tensile strength but also eliminates the positive effect of different heating rates.

So far, several works have focused on the relationship between structural evolution and properties as the key role to improve mechanical performance^5^. The current opinion is that a combination of a low heating rate and a high carbonization temperature render more energy and time to develop a more ordered structure from either the amorphous phase or the basic structural units (BSU) as defined by Deurbergue and Oberlin^3^. These conditions would allow to densify the stacked layers during the carbonization, the process where atoms different from carbon are removed, which involves gas formation. However, our Raman spectroscopy and XRD results rule out this historically built hypothesis, as we did not find a significant difference in crystallite size and orientation in the present experiments.

We carried out the elemental analysis of the samples to understand the chemical evolution of the CFs as a function of both temperature and heating rate (Figure S7). The chemical compositions clearly indicate an enrichment of the carbon fraction when the temperature increases, as expected. During carbonization, there is a mass reduction process of the fibers, where H_2_O, HCN and N_2_ evolve, and consequently, the carbon fraction of the fibers increases with a simultaneous decrease of the nitrogen and oxygen content. This trend was also observed by XPS measurements, although the atomic percentages between both techniques vary due to the technical differences.

Four peaks were proposed from the deconvolution of the N 1s peak (Figure S9, Table S1): pyridinic, pyridonic/pyrrolic, quaternary and oxidized nitrogens, with binding energies at 398.50, 401.05, 401.77, and 403.28 eV, respectively^18^,^19^. The amount of pyridinic N, pyridonic/pyrrolic N and oxidized N increased, but the amount of quaternary N decreased with an increase of the heating rate regardless of the carbonization temperature. The quaternary nitrogen fraction becomes a maximum at the carbonization temperature of 1050 °C. Furthermore, from the C 1 s deconvolution, we observed that the amount of sp^3^ shows a similar trend to that of quaternary nitrogen atoms in CFs. Additionally, lowering the heating rate induced higher sp^3^ content, resulting in higher tensile strength even though the carbonization temperature is different (Figs 1e, S10 and Table S2). At 1200 °C, these effects are not significant and the tensile strength do not contribute to the trend lines. It has been reported that the modulus of the carbon fiber increases with the decrease of sp^3^ content^20^,^21^,^22^,^23^. However, to the best of our knowledge, there is no report on a correlation between tensile strength and the chemical structure, particularly, sp^3^ and quaternary N contents.

CFs carbonized at 1200 °C have less nitrogen on the edge of the basal planes of the crystalline structure which results in more dangling bonds and leading to a higher conductive carrier concentration, as well as a higher carrier mobility^24^. However, a lower amount of nitrogen does not help in enhancing tensile strength, and a lower heating rate of CFs higher tensile strength Fig 1d). CFs heated to 1050 °C at 0.5 °C/min showed the highest sp^3^ contents (Fig 1f), as described above, as described above. Additionally, the pyridinic nitrogen content for 1050 °C and 0.5 °C/min is lower than that under any other heating condition. This suggests that the lowest content of pyridinic nitrogen in the CFs is always associated with vacancies^25^,^26^. Therefore, vacancies should also disappear with increasing van der Waals attraction between layers, enhancing the mechanical properties.

Deurbergue and Oberlin first recognized the role of nitrogen atoms in the carbon structure of pyrolized PAN in 1991^3^. They reported that low pyrolysis rates lead to a high nitrogen content, which presumably could be retained during the rearrangement of carbon atoms until the later stage of carbonization. The proposed mechanism consisted of nitrogen being located in aromatic rings bonded together across the graphitic planes, thus increasing the density of the carbon and consequently improving the tensile strength. The different chemical environments of the nitrogen atoms were not clear, but it was suggested that these atoms were possibly located at the edges of the graphitic planes forming dangling bonds that prevented the coalescence of carbon networks^27^. The absence of coalescence would preserve the small size of the crystalline layers, which contributed to the fine structure and high strength of the material.

Based on these results, which show a strong correlation between the strength properties and the amount of quaternary nitrogen atoms and sp^3^ bonding in the CFs, we conducted *ab initio* calculations to understand the role of quaternary nitrogen atoms and sp^3^ bonding in the tensile strength of CFs. Such nitrogen atoms contained in carbon networks have not shown a significant proximity in terms of the interlayer spacing, which can increase the strength due to the strengthened van der Waals force. Additionally, no occurrence of sp^3^ bonds was obtained under approximately 1000 °C. Therefore, we focused on the phenomenon of interstitial carbon atoms in the interlayer space of the nitrogen-containing carbon networks. Interstitial carbon atoms were clearly observed by HR-TEM between the two shells of double walled carbon nanotubes^28^. This observation was supported by *ab initio* calculations of the sp^3^ bonding between the inner and outer tubes in the double-wall carbon nanotube, which affected the mechanical properties^29^.

**Figure 2:**
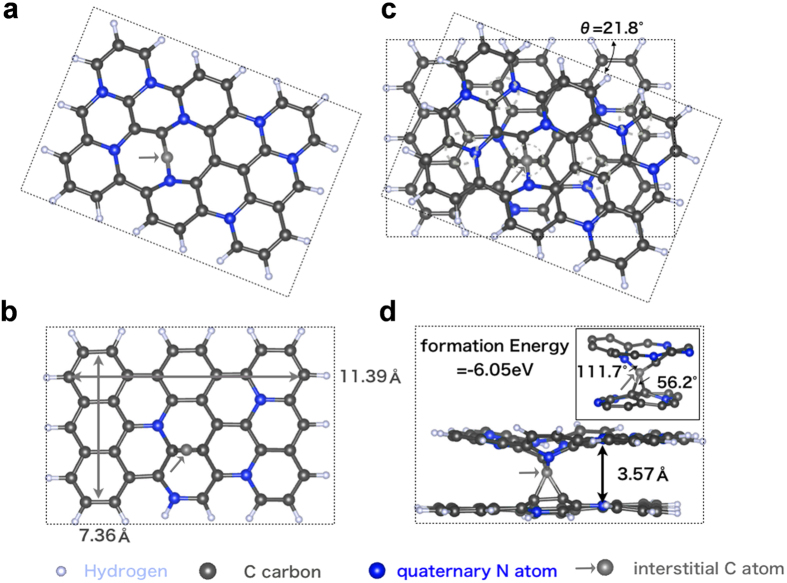
Simulated stacking arrangements of the hexagonal carbon network including quaternary nitrogen atoms with an interstitial carbon atom forming a stable sp^3^ bonding between the two layers. (**a**)Top view of the turbostratic stacking of the upper layer with an interstitial carbon atom, (**b**) top view of the turbostratic stacking of the lower layer with an interstitial carbon atom, and (**c**) turbostratic stacking in a bilayer. Any circle colored in aqua denotes a point with the potential of cross-linking between the adjacent carbon layers. (**d**) The interstitial carbon atom generates the sp^3^ hybridization binding with an interlayer spacing of 3.57 Å. Here, the C−C−C angle at the lower layer and the N−C−C angle at the upper layer are 56.2° and 111.7°, respectively.

Figure 2 shows the turbostratic structure obtained after geometry relaxation with hydrogen termination in our model. Only the upper and lower layer with the interstitial C atom are shown in Fig. 2a,b, respectively. In order to understand the effect of quaternary N atoms and interstitial C atoms, we calculated the interlayer distance and binding energy for the two structures: (a) and (b) as references. The first structure, (c), is a stacked bilayer structure of pure carbon. For this case, the vdW interaction between layers keeps the interlayer distance at 3.87 Å. This is relatively larger than the typical value of turbostratic carbon (3.40 Å), presumably because of a smaller number of stacked layers that give rise to a weaker vdW interaction. The binding energy of this structure was defined as ΔE=(E_up + E_down)-E_bi. Here, E_up, E_down, and E_bi are the total energies for the upper layer, lower layer and combined bilayer with an optimized distance, respectively. The resulting binding energy is 1.83 eV. Note that the larger binding energy the structure has, the more energetically stable the structure is. The pure carbon bilayers become more stable than the two isolated carbon layers.

The second structure, (b), is a stacked bilayer structure doped with nitrogen. The C atoms in the upper and lower layers of the (a) structure were replaced by 8 and 4 nitrogen atoms, on the quaternary site, respectively (see Fig. 2a,b without the interstitial C atom). The relaxation shows that the interlayer distance of the stacked bilayer with quaternary N atoms is 3.51 Å, which is smaller than that of pure carbon by a difference of 0.36 (3.87–3.51) Å. The attractive interlayer vdW interaction increased because of the electron-rich nitrogen atoms. After the inclusion of nitrogen atoms, the binding energy was 2.07 eV, which is larger than that of the bilayer with pure carbon. The result is consistent with the interlayer distance results determined by the strength of binding energy.

In Fig. 2c,d, besides the quaternary N atoms, we intercalated carbon atoms between bilayers with our modeling during the carbonization process. These atoms can generate sp^3^ hybridization binding between the turbostratic bilayers. This sp^3^ hybridization easily occurs if the layer arrangement is similar to our structure modeling with a twisted angle of θ=21.8° in the turbostratic stacking. In addition, the interlayer distance became shorter with the quaternary N atoms, and sp^3^ hybridization through the interstitial C atom also occurred easily. The simulation result of the interlayer distance of this structure is 3.57 Å, which is slightly larger than 3.51 Å without interstitial carbon atoms due to atomic volume spacing. The resultant calculated interlayer spacing is within a similar range as the one measured for the interlayer spacing d_002_ of 3.50–3.60 Å in PAN-based CFs. The binding energy of the structure with quaternary N atoms and interstitial C atoms was defined as ΔE=(E_C + E_bi)-E_sp^3^. Here, E_sp^3^ is the total energy for the structure (Fig. 2d) with sp^3^ hybridization. The resulting binding energy is 6.05 eV. The result shows the effect of the existence of interstitial carbon atoms in the interlayer space. The interstitial carbon atoms form cross-linking by sp^3^ bonding, which makes a stable turbostratic structure and causes a strength enhancement.

**Figure 3:**
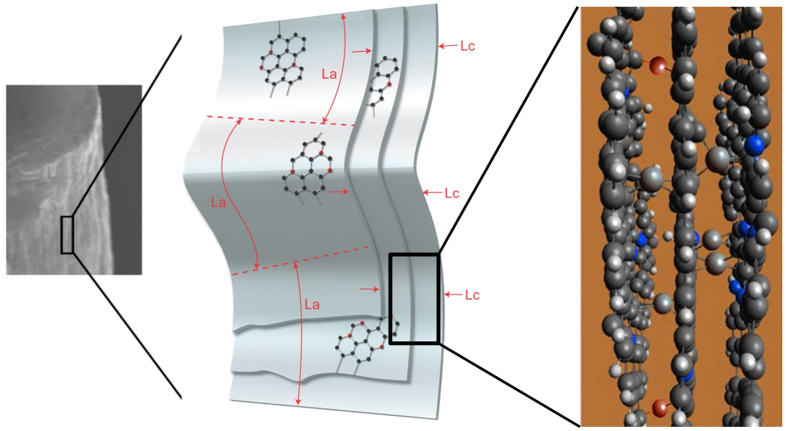
PAN-based carbon fiber model showing the quaternary nitrogen atoms in the carbon hexagonal networks (blue) and the proposed sp^3^ bonding between the stacked layers mediated by the interstitial carbon atoms located in the interlayer space. Oxygen crosslinking atoms are shown in red.

It is evident that during this process, the nitrogen atoms in the hexagonal carbon networks with quaternary bonding can increase the stability to establish sp^3^ bonding with the introduction of interstitial carbon atoms, which promotes the mechanical strength by a cross-linking of the adjacent carbon layers. The stability of this kind of arrangement is supported by previous reports in the literature in pure carbon layers, where interstitial carbon atoms are expected to increase the resistance to crack propagation between layers in graphite, similarly to a composite^30^,^31^. Indeed, a similar phenomenon has been seen in neutron-irradiated graphite.^32^ The reinforcing effect of the interstitial carbon between graphitic layers should be higher than that provided by oxygen cross linking sites, due to the presence of four bonds, instead of two. It is interesting that more nitrogen atoms with quaternary bonding increased the instability. To estimate the mechanical contribution of quaternary N atoms and interstitial C atoms, the binding energy in each case was calculated (Table S3). It is known that by establishing the sp^3^ structure, the structural effect evidently contributes to the mechanical performance. By combining the traditionally established structural model of PAN-based CFs, a new model based on the present result is proposed as shown in Fig. 3.

## Discussion

In conclusion, the key factors to achieve high-strength PAN-based CFs were investigated by using commercially available PAN precursor fibers and carbonizing them at different temperatures and exploring several heating rates. We found that nitrogen atoms with quaternary bonding in the hexagonal carbon networks and sp^3^ bonding clearly increased the tensile strength performance of the CFs. Such structures of CFs rich in nitrogen and sp^3^ were achieved by using a low heating rate. These nitrogen atoms support the formation of sp^3^ bonding between the turbostratic carbon layers. We carried out geometry relaxation of the interstitial carbon atoms located between the stacked carbon basal planes during carbonization. These cross-linked carbon layers are expected to contribute to the higher strength that was experimentally found. These results provide new information towards the conventional theory in which the highest carbonization temperature is the most important parameter to determine the structure and properties of PAN-based CFs. Additionally we used a commercial-grade PAN precursor that reduces the cost of PAN-based CFs production. These results provide another pathway to further improve the tensile strength performances of CFs.

## Additional Information

**How to cite this article**: Kim, M.-A. *et al*. Strengthened PAN-based carbon fibers obtained by slow heating rate carbonization. *Sci. Rep.* **6**, 22988; doi: 10.1038/srep22988 (2016).

## Supplementary Material

Supplementary Information
