# Supplementary material for: Strengthened PAN-based carbon fibers obtained by slow heating rate carbonization
Source: Sci Rep. 2016 Mar 23;6:22988. doi: 10.1038/srep22988 (PMC4804295; doi:10.1038/srep22988)
Supplement: Supplementary Information [file srep22988-s1.doc]

Supplementary Information

**Strengthened PAN-based carbon fibers obtained by slow heating rate carbonization**

Min-A Kim^1,2^, Dawon Jang^1,3^, Syogo Tejima^4,6^, Rodolfo Cruz-Silva^4^, Han-Ik Joh^1,3^,

Hwan Chul Kim^2^, Sungho Lee^1,3*^, and Morinobu Endo^4,5*^

^1^Carbon Convergence Materials Research Center,

Korea Institute of Science and Technology

San 101 Eunha-ri, Bongdong-eup, Wanju-gun, Jeonbuk 565-905, Korea.

^2^Department of Organic Materials and Fiber Engineering,

Chonbuk National University, 567 baeje-daero, Deokjin-gu, Jeonju, Jeonbuk 561-756, Korea.

^3^Department of Nanomaterials Engineering, Korea University of Science and Technology,217 Gajeong-ro, Tuseong-gu, Daejeon, 305-350 Korea.

^4^Global Aqua Innovation Center, Shinshu University, 4-17-1 Wakasato, Nagano 380-8553, Japan.

^5^Institute of carbon science and technology, Shinshu University, 4-17-1 Wakasato, Nagano 380-8553, Japan.

^6^Research Organization for Information Science & Technology, 2-32-3, Kitashinagawa, Shinagawa-ku, Tokyo, 140-0001.

*e-mail: [sunghol@kist.re.kr](mailto:sunghol@kist.re.kr); endo@endomoribu.shinshu-u.ac.jp

Table S1. N1s deconvolution results.

| Temperature  ℃ | Heating rate  ℃/min | Composition (% of total nitrogen) | | | |
| --- | --- | --- | --- | --- | --- |
|  |  | Pyridinic | Pyridonic/pyrrolic | Quaternary | Oxidized |
| 1000 | 0.5 | 42.1 | 35.8 | 6.9 | 15.2 |
|  | 1 | 43.0 | 37.0 | 5.1 | 14.9 |
|  | 2 | 45.0 | 37.0 | 4.1 | 14.0 |
|  | 5 | 49.1 | 34.9 | 2.8 | 13.3 |
|  | 10 | 49.4 | 35.3 | 2.1 | 13.2 |
| 1050 | 0.5 | 26.2 | 40.1 | 16 | 17.7 |
|  | 1 | 32.8 | 36.6 | 12.7 | 17.9 |
|  | 2 | 34.4 | 37.1 | 9.7 | 18.7 |
|  | 5 | 43.8 | 33.2 | 6.0 | 16.9 |
|  | 10 | 40.2 | 40.1 | 4.8 | 14.9 |
| 1100 | 0.5 | 23.1 | 43.8 | 14.1 | 19.0 |
|  | 1 | 25.1 | 44.7 | 10.5 | 19.8 |
|  | 2 | 31.9 | 42.0 | 9.0 | 17.0 |
|  | 5 | 36.6 | 41.9 | 6.4 | 15.1 |
|  | 10 | 38.8 | 39.3 | 5.0 | 16.8 |
| 1200 | 0.5 | 21.4 | 42.5 | 11.2 | 24.9 |
|  | 1 | 20.5 | 44.2 | 9.7 | 25.6 |
|  | 2 | 21.1 | 42.5 | 7.6 | 28.8 |
|  | 5 | 24.3 | 41.2 | 7.0 | 27.5 |
|  | 10 | 26.9 | 47.0 | 4.7 | 21.5 |

Table S2. C1s deconvolution results.

| Temperature  ℃ | Heating rate  ℃/min | Composition (% of total carbon) | | | | |
| --- | --- | --- | --- | --- | --- | --- |
|  |  | Carbon  sp^2^ | Carbon  sp^3^ | Epoxy/hydroxyl groups (C-O)  /C-N | Carbonyl group (C=O) | Carboxyl group  (O-C=O) |
| 1000 | 0.5 | 49.5 | 21.2 | 13.5 | 12.0 | 3.0 |
|  | 1 | 49.0 | 20.6 | 14.3 | 12.3 | 3.8 |
|  | 2 | 47.8 | 19.3 | 15.6 | 13.4 | 3.9 |
|  | 5 | 47.5 | 17.3 | 16.5 | 14.4 | 4.2 |
|  | 10 | 47.4 | 15.5 | 17.0 | 15.5 | 4.5 |
| 1050 | 0.5 | 51.4 | 23.9 | 9.6 | 10.1 | 5.1 |
|  | 1 | 51.1 | 22.7 | 10.0 | 10.4 | 5.7 |
|  | 2 | 51.0 | 21.9 | 10.3 | 11.0 | 6.0 |
|  | 5 | 49.6 | 21.2 | 10.9 | 12.1 | 6.2 |
|  | 10 | 48.7 | 19.4 | 11.2 | 13.8 | 6.9 |
| 1100 | 0.5 | 56.2 | 19.9 | 9.0 | 9.5 | 5.4 |
|  | 1 | 53.1 | 20.0 | 10.4 | 10.6 | 5.8 |
|  | 2 | 51.6 | 20.0 | 10.8 | 11.3 | 6.4 |
|  | 5 | 49.7 | 20.0 | 11.4 | 12.2 | 6.7 |
|  | 10 | 49.6 | 18.7 | 11.9 | 12.5 | 7.3 |
| 1200 | 0.5 | 55.0 | 19.1 | 9.7 | 10.9 | 5.2 |
|  | 1 | 53.2 | 18.8 | 10.8 | 11.8 | 5.4 |
|  | 2 | 51.0 | 18.6 | 12.0 | 12.3 | 6.2 |
|  | 5 | 48.3 | 18.6 | 12.5 | 14.1 | 6.6 |
|  | 10 | 45.3 | 18.6 | 15.0 | 14.3 | 6.7 |

Table S3. Interlayer distance and formation energy for three types of turbostratic structures

| Characteristic of structure | Interlayer distance (Å) | Formation energy( eV) |
| --- | --- | --- |
| ・hexagonal C atom | 3.87 | -1.83 |
| ・hexagonal C atom  ・Quaternary N atom | 3.51 | -2.07 |
| ・hexagonal C atom  ・Quaternary N atom  ・Interstitial C atom | 3.57 | -6.05 |


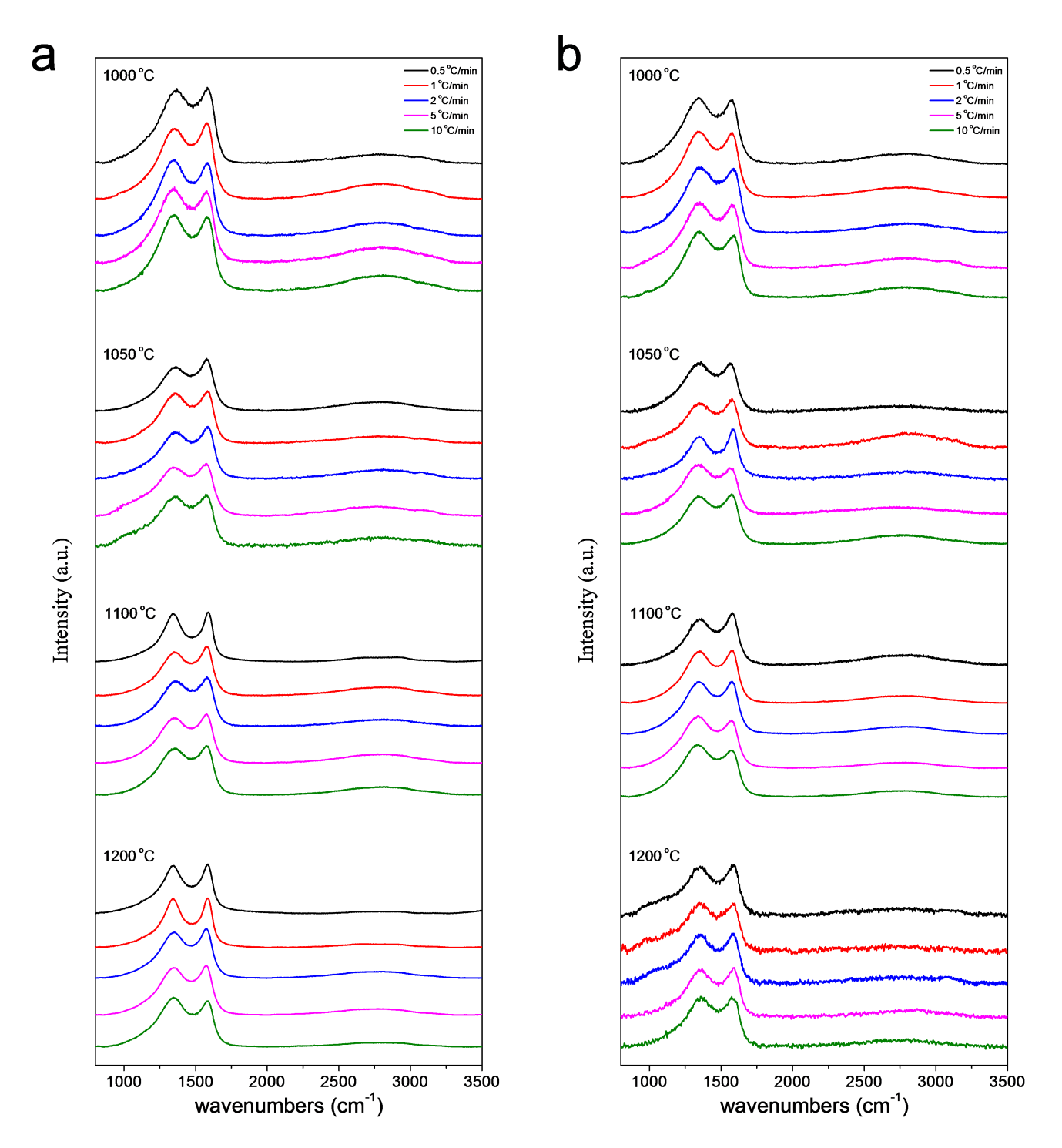


Figure S1. Raman spectra of carbon fiber heated by different conditions, where the incident beam is (a) parallel and (b) perpendicular to the fiber axis.


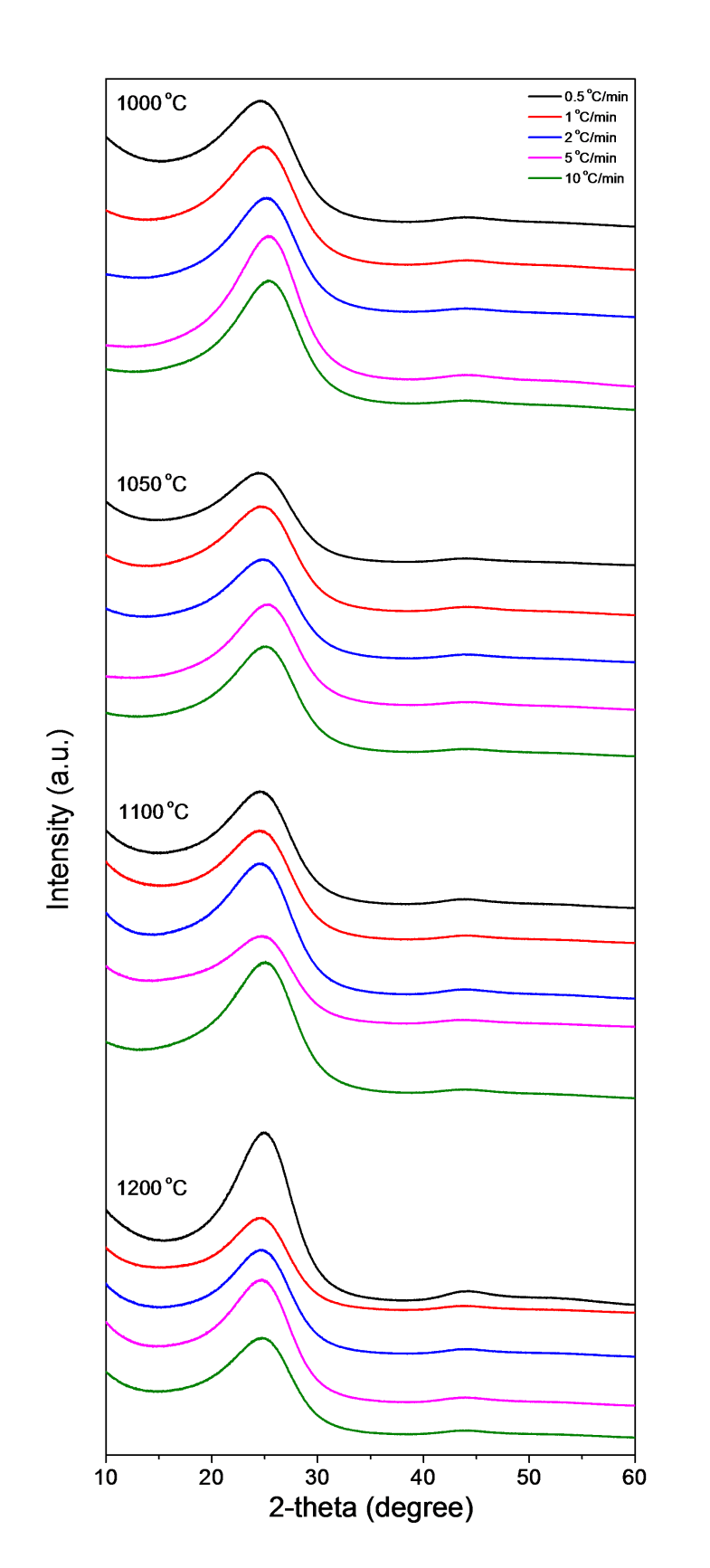


Figure S2. Radial (2θ) intensity profiles of carbon fibers heated by different conditions, where X-ray is irradiated perpendicular to the fiber axis.


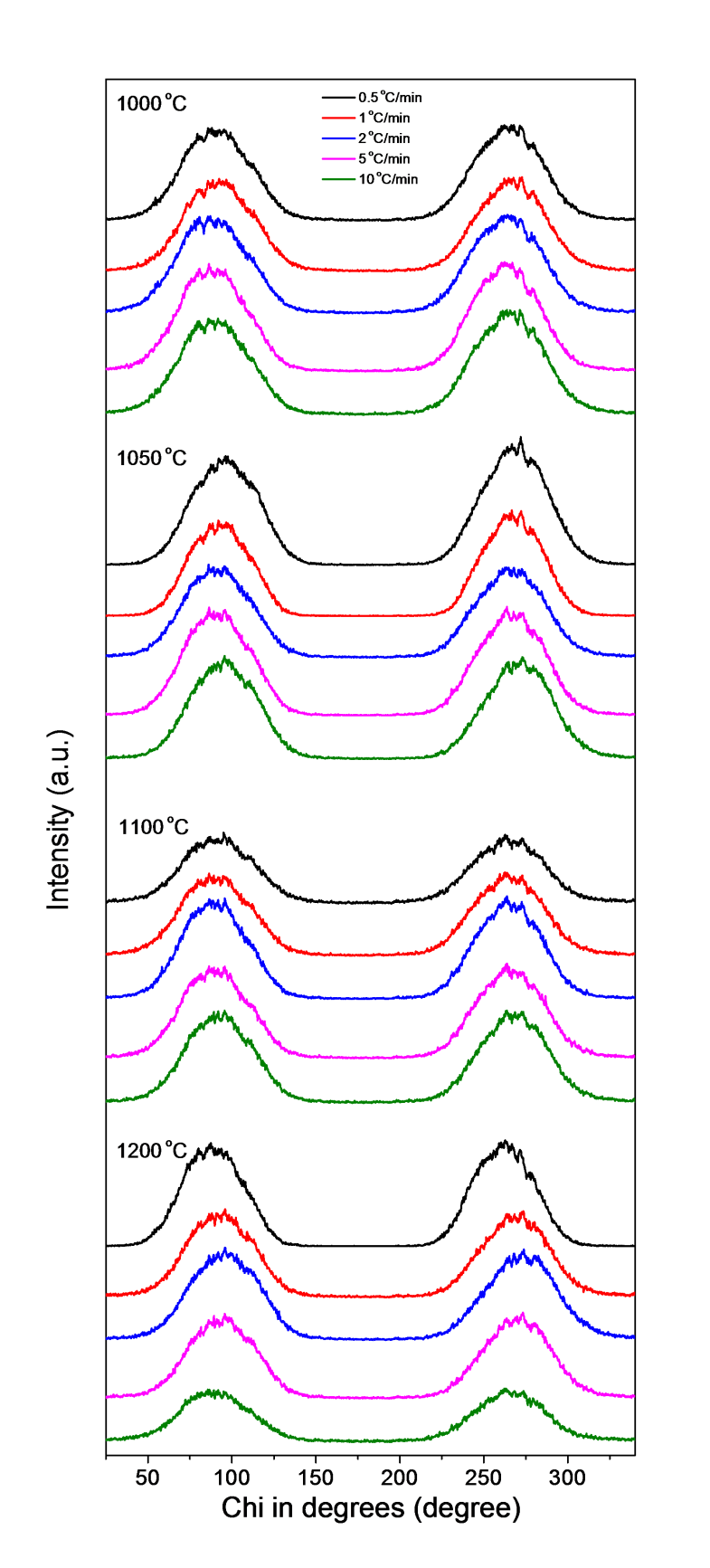


Figure S3. Azimuthal intensity profiles of carbon fibers heated by different conditions, where X-ray is irradiated perpendicular to the fiber axis.


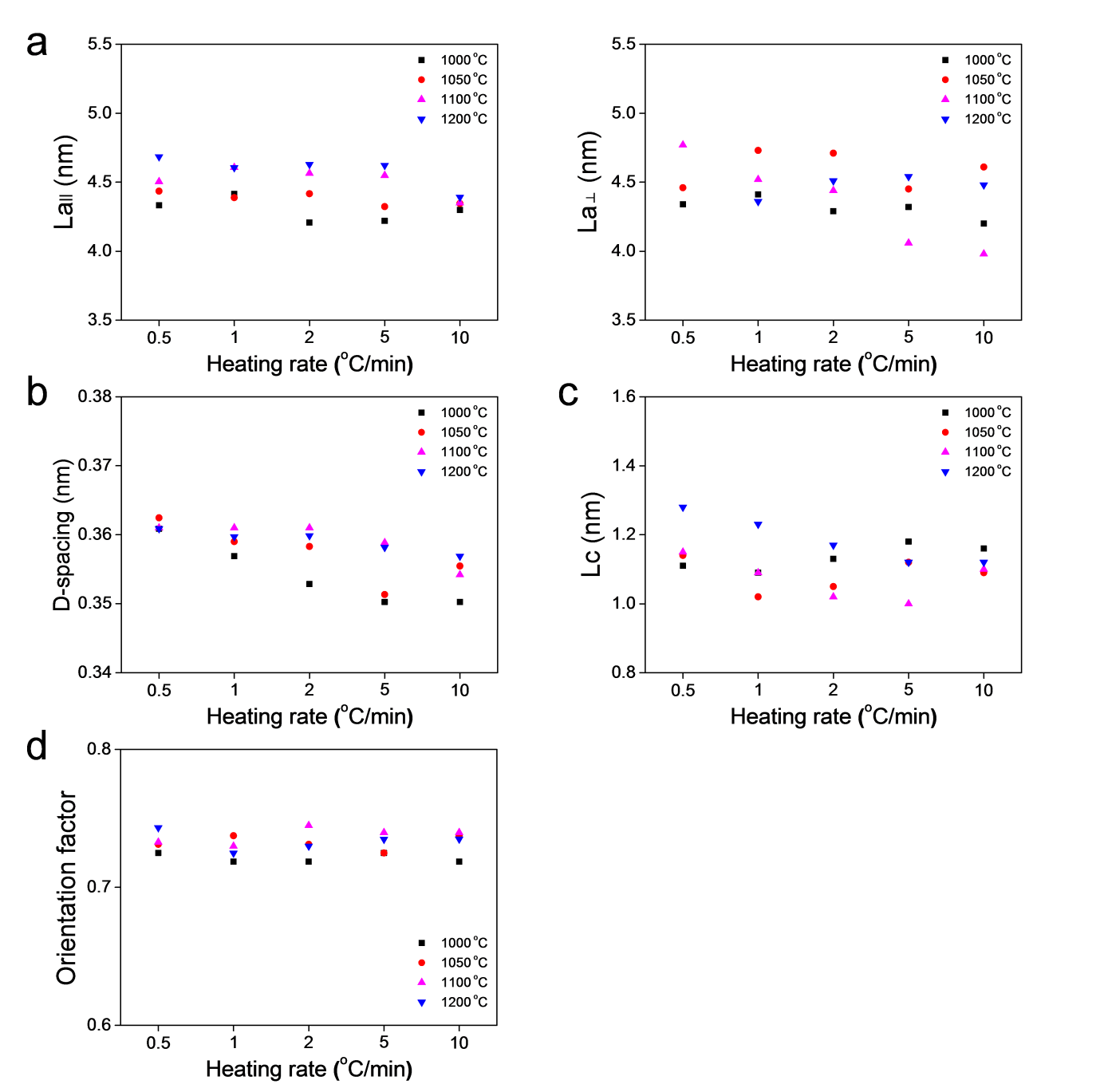


Figure S4. The structural parameters of carbon fibers from Raman spectroscopy and X-ray diffraction: (a) layer length consisting of carbon hexagonal network, (L_a_) to parallel and perpendicular to the fiber axis, (b) the interlayer spacing d_002_ at the (002) plane, (c) the parallel stacking height of the carbon hexagonal network (L_c_), and (d) the orientation factor of carbon fibers depending on heating conditions.

**
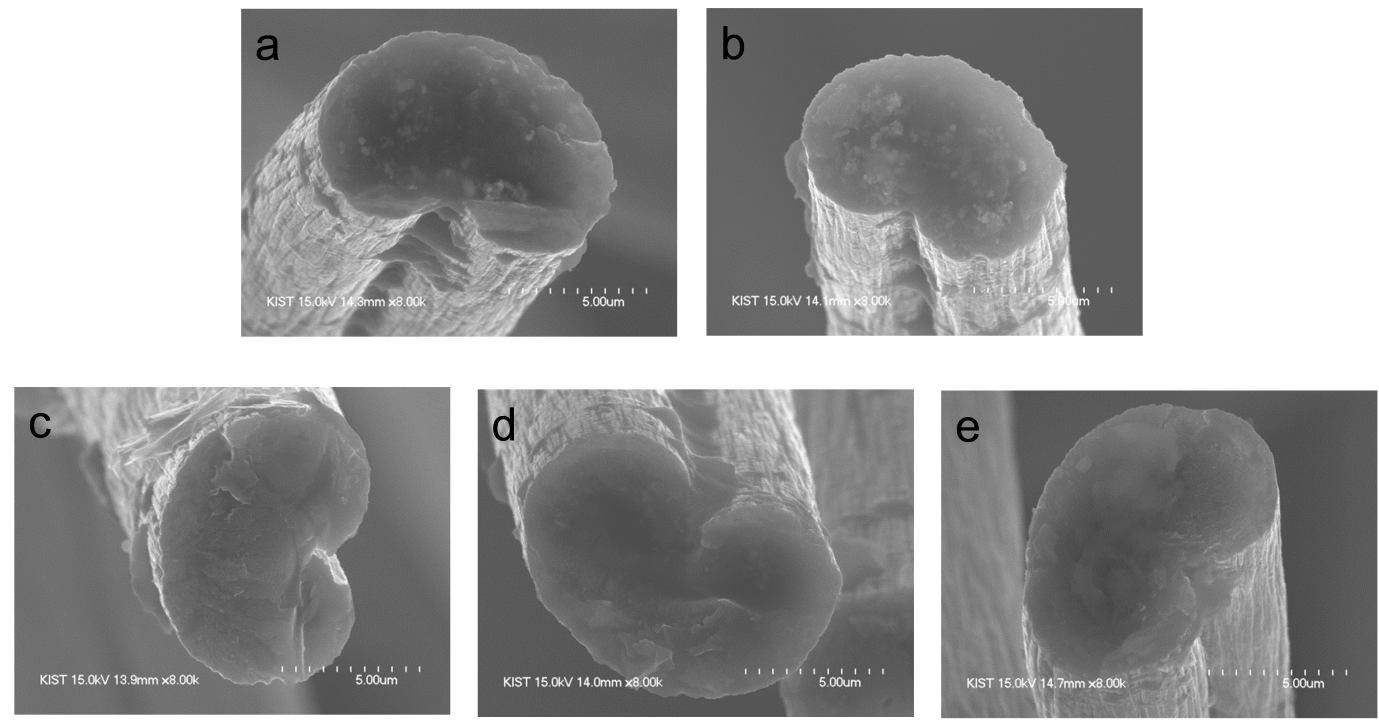
**

Figure S5. SEM images of carbon fiber heated at 1050 ℃ with (a) 0.5, (b) 1, (c) 2, (d) 5, and (e) 10 ℃/min heating rates.


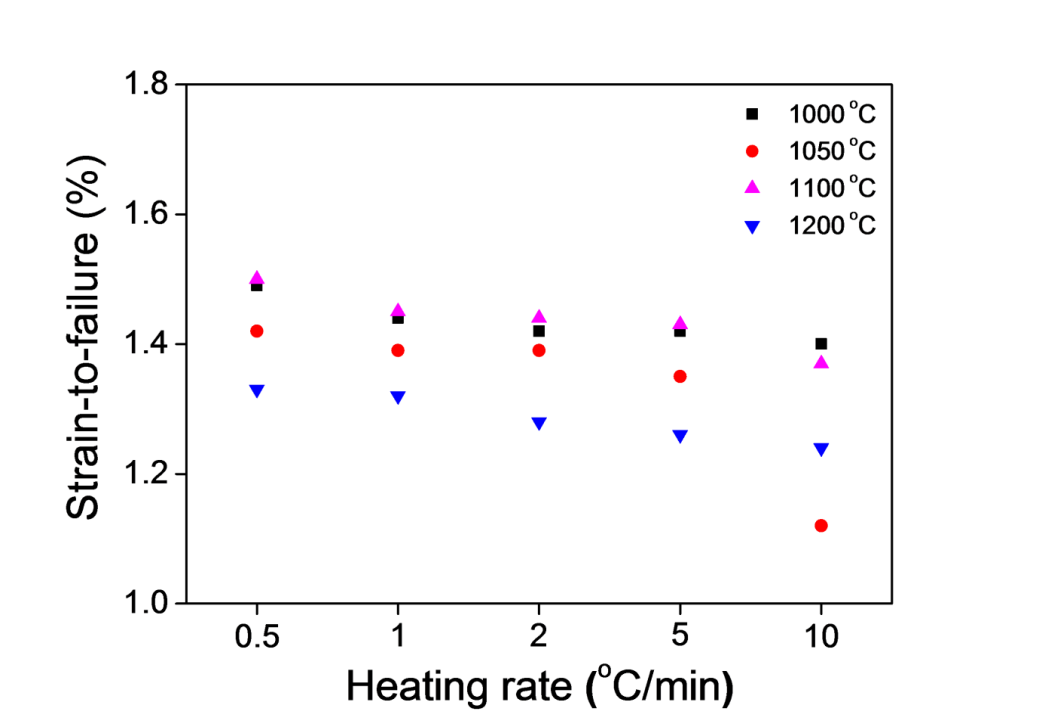


Figure S6. Strain-to-failure of carbon fiber as a function of the heating rate at 1000, 1050, 1100, and 1200 ℃, respectively.


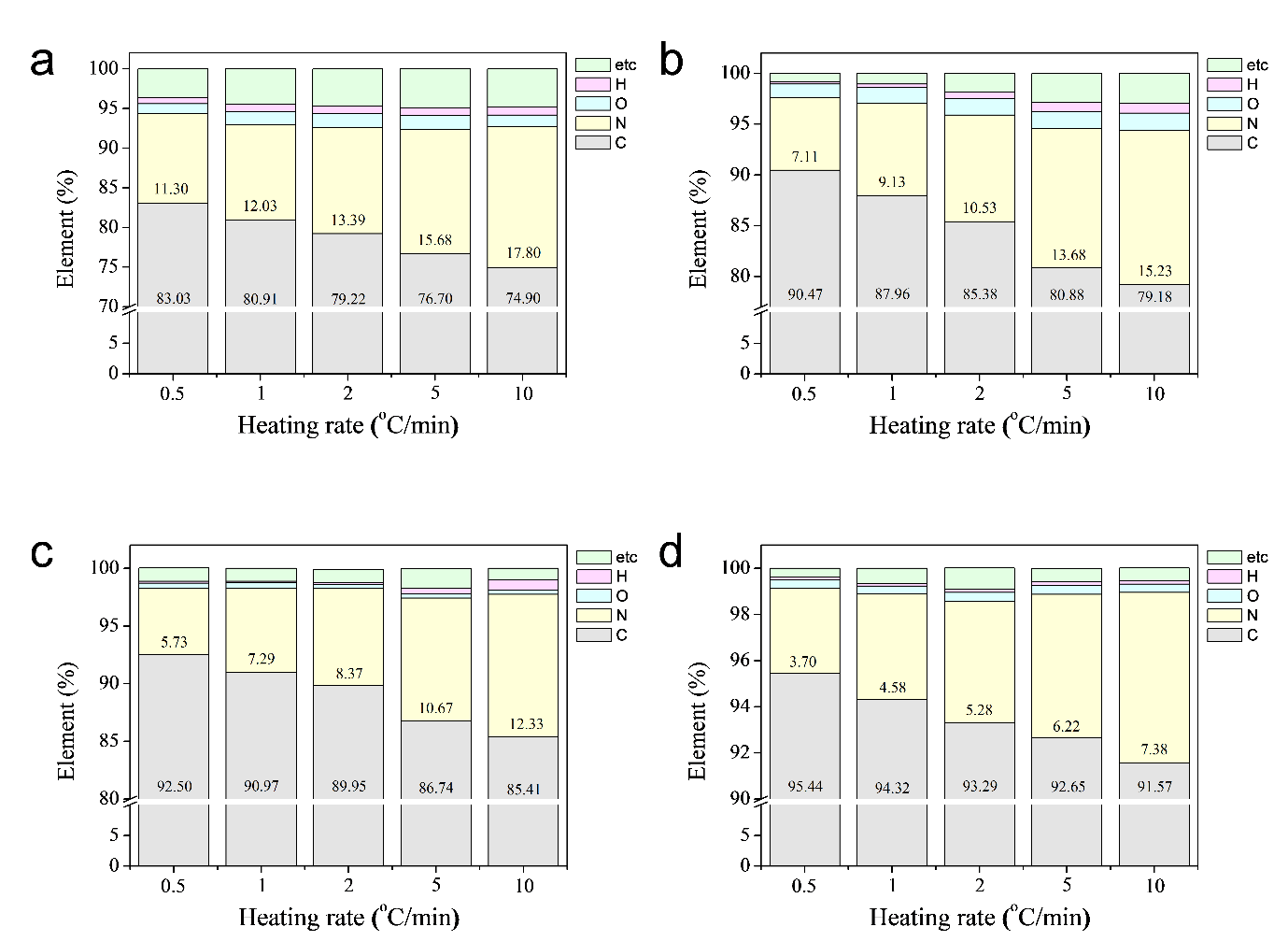


Figure S7. Elemental analysis of carbon fiber heated at (a) 1000 ℃, (b) 1050 ℃, (c) 1100 ℃, and (d) 1200 ℃ with different heating rates (0.5 - 10 ℃/min).

**
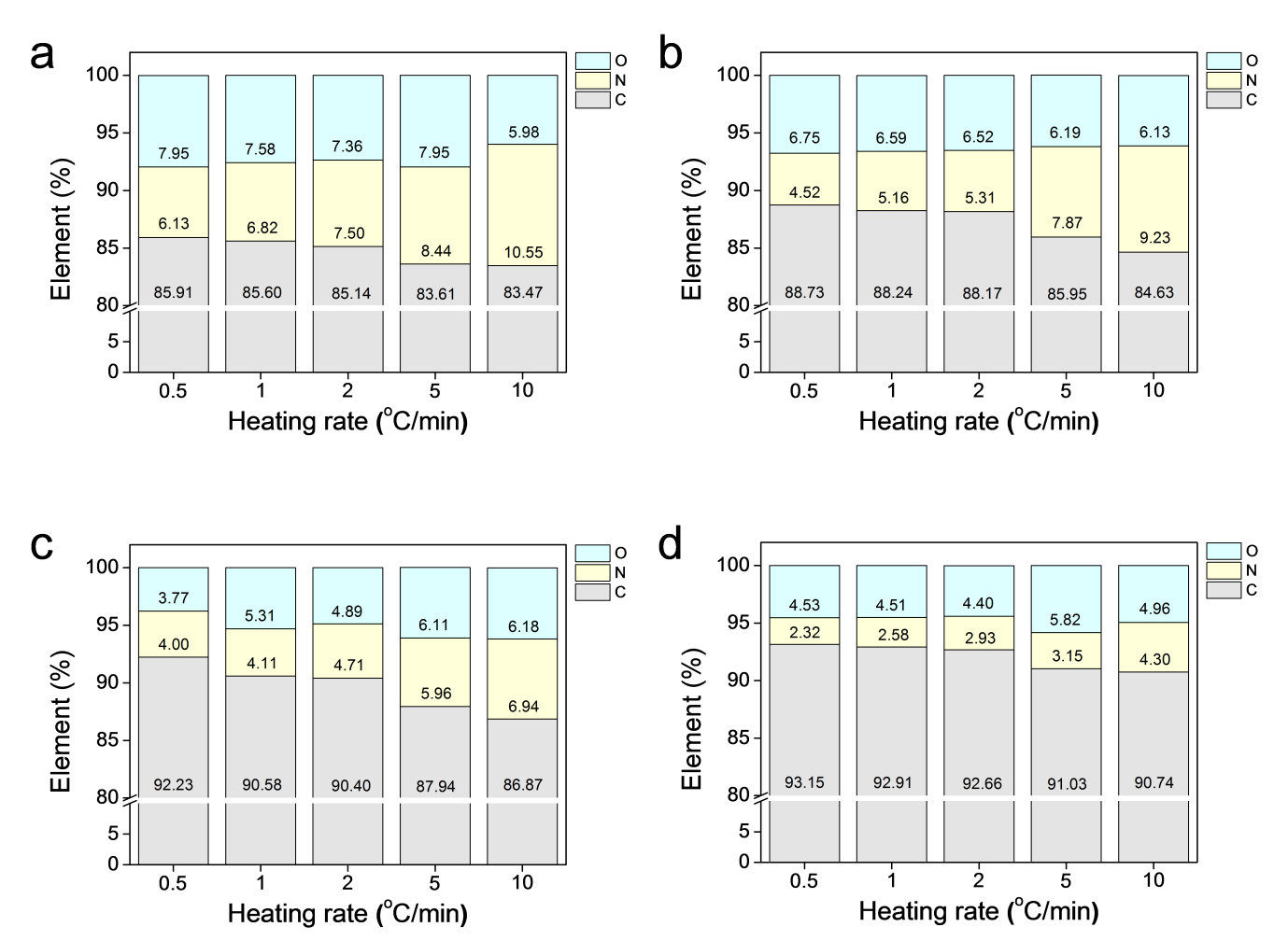
**

Figure S8. Semiquantitative XPS analysis of carbon fiber heated at (a) 1000 ℃, (b) 1050 ℃, (c) 1100 ℃, and (d) 1200 ℃ with different heating rates (0.5 - 10 ℃/min).


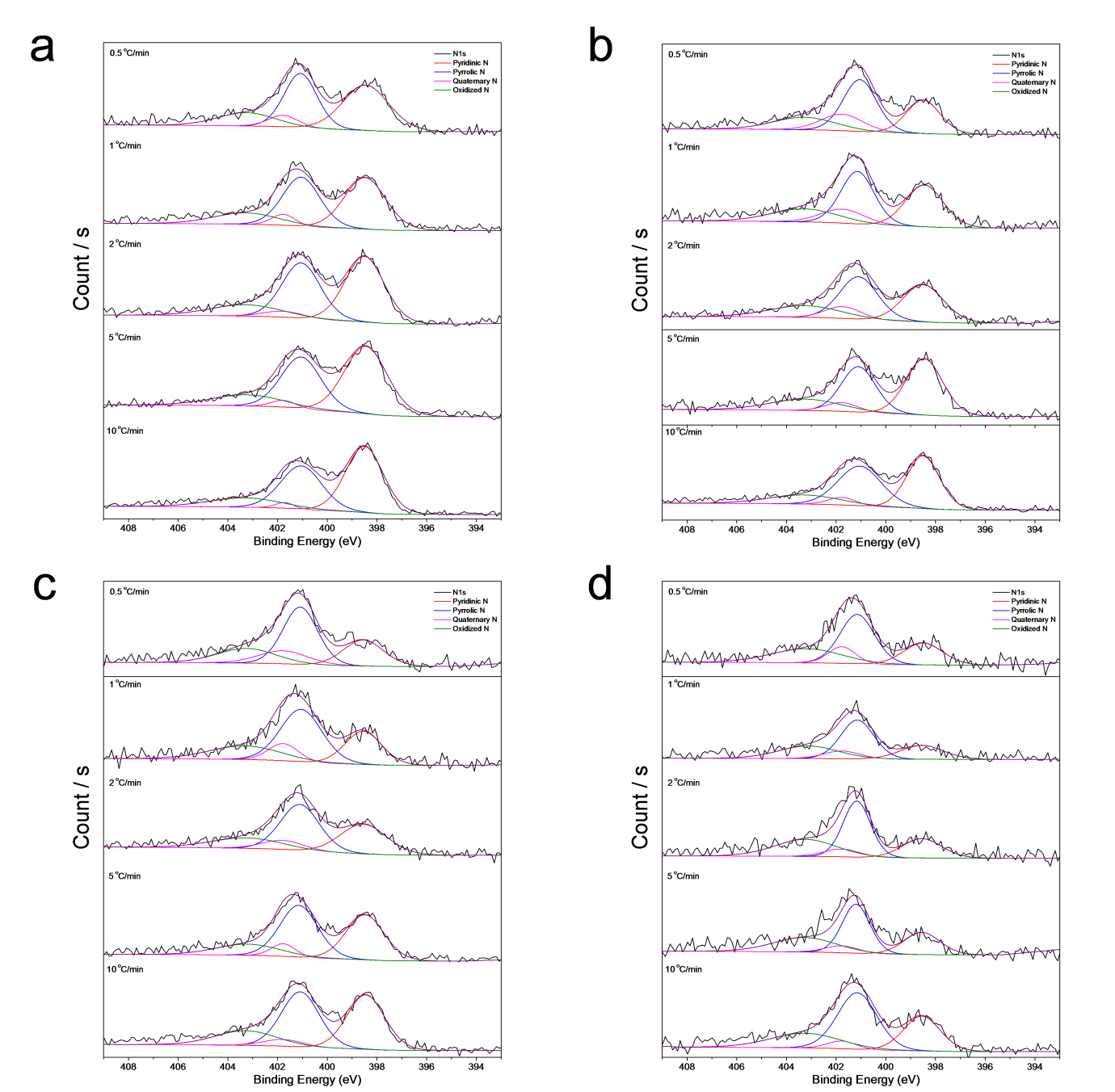


Figure S9. N1s deconvolution spectra of carbon fiber heated by different conditions: up to carbonization temperature of (a) 1000 ℃, (b) 1050 ℃, (c) 1100 ℃ and (d) 1200 ℃, respectively.


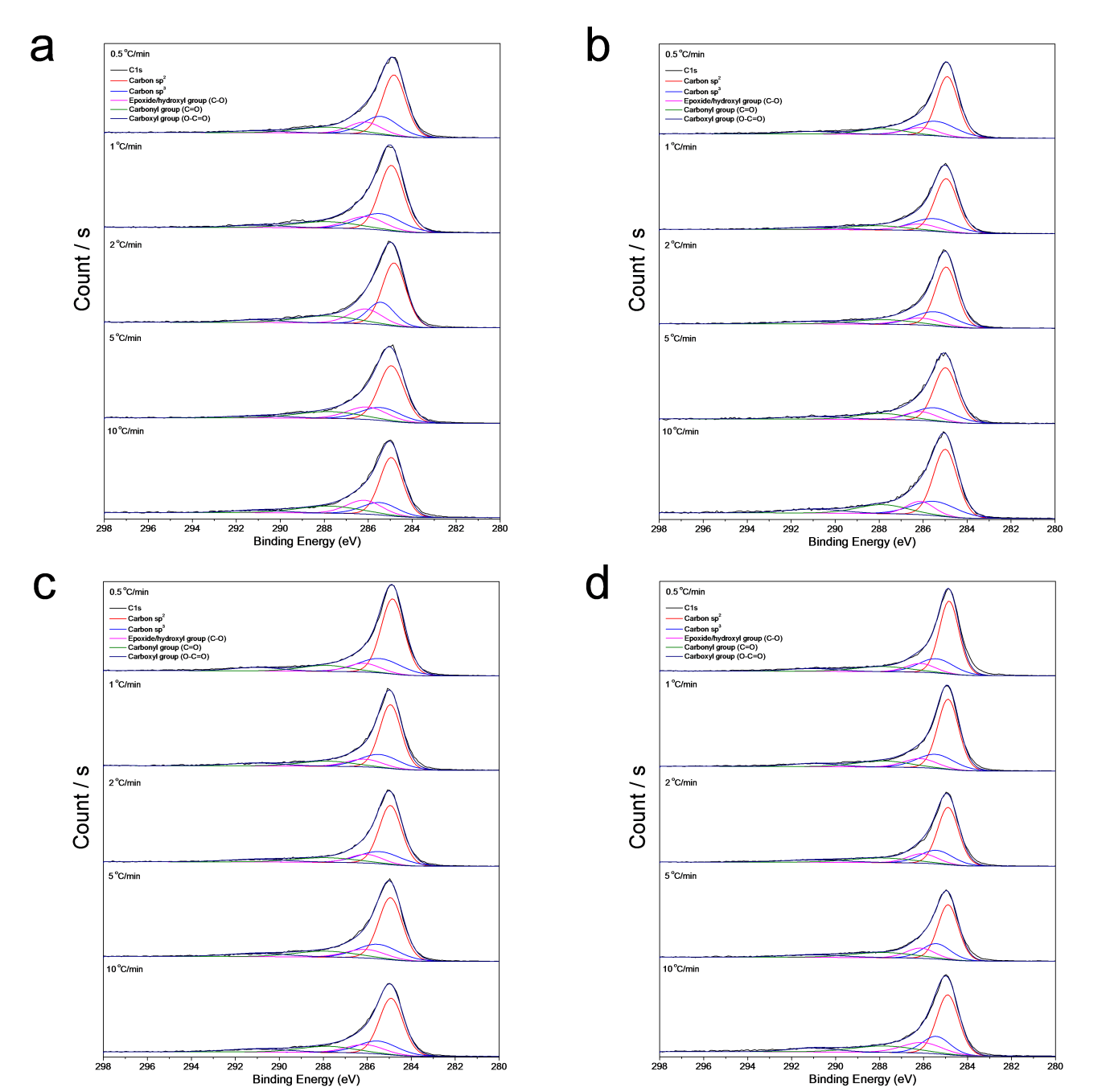


Figure S10. C1s deconvolution spectra of carbon fiber heated by different conditions: up to carbonization temperature of (a) 1000 ℃, (b) 1050 ℃, (c) 1100 ℃ and (d) 1200 ℃, respectively.
